# Supplementary material for: Physical and financial access challenges to seeking child healthcare in a rural district in Ghana
Source: PLoS One. 2025 Apr 16;20(4):e0321768. doi: 10.1371/journal.pone.0321768 (PMC12002520; doi:10.1371/journal.pone.0321768)
Supplement: Attachment — Submitted filename: Response to reviewers.docx [file pone.0321768.s002.docx]

**AUTHORS’ RESPONSE TO REVIEW COMMENTS**

**Journal:** Plosone

**Manuscript title:** Physical and financial access challenges to seeking child healthcare in a rural district in Ghana.

**Authors:** Felix Kwasi Nyande, Esmeralda Ricks, Margaret Williams, and Sihaam Jardien-Baboo

**Manuscript number**: PONE-D-24-14484

We have considered the reviewers’ comments and have incorporated changes accordingly into the revised version.

| **s/n** | **Review comment** | **Response** |
| --- | --- | --- |
| **Reviewer #1** | | |
| **1** | The manuscript is very well written.  -methodology part has clarity in terms of how the participants were recruited, how data recorded and analyzed. Particularly use of criterion of credibility, dependability, transferability, and confirmability is important that many manuscripts lack. | thank you for the comments  Comment well appreciated |
| **2** | -analysis is presented in a manner that suitably answers the study objectives. only thing I would ask authors is to include a table listing codes that emerged during data analysis. Including such detail will further strengthen the manuscript as readers would be able to see how codes led to emergence of themes. | A table listing all the codes that emerged during the data analysis has been included as table 1 |
| **3** | -discussion is quite detailed comparing findings with those reported in literature. | Thank you for your comments |
| Reviewer #2 | | |
| **1** | INTRODUCTION 1. The words aimed to develop abbreviations should all be in capital letter e.g. LMICs in line 66 is sourced from lowercase letters. This also applies to similar cases within this article. | The words aimed to develop abbreviations have been capitalised throughout the manuscript |
| **2** | In literature review, most of the studies are locally by origin. Thus, they are mostly from Ghana. It will be better if some studies out of Ghana are added to this work to put this study in international context. | Studies from outside Ghana have been included in the literature review to put the work in international context. |
| **3** | The physical and financial aspects can be summarized to be access. You may think of improving your objective and tittle to be more inclusive by mentioning of access to include both physical and financial aspects. | Access to healthcare is a relatively complex multidimensional phenomenon. This manuscript only considered the physical and financial dimensions excluding other dimensions. Since the dimensions to healthcare access go beyond the physical and financial, there is the need to be specific with the dimensions considered in this manuscript. |
| **4** | The presentation of core aspects of this study presents serious inconsistency in writing. For instance, in abstract the objective is “To explore the experiences of nurses and caregivers about the physical and financial 30 challenges to accessing child healthcare services in a rural district in Ghana” while in main article is “experiences of nurses and caregivers about accessibility challenges that impeded access to child healthcare services”. This is further written differently in line 81-82 presenting a slightly different phrase. In line 189, there is completely different thing but aiming on the same meaning. While they might be closely related, they are not the same. The core message should be consistent across the article. | The inconsistencies observed with the stating of the objective of the study have been corrected throughout the manuscript. |
| **5** | METHODOLOGY There are five qualitative designs including Ethnography, Narratives, Grounded theory, Phenomenology and Case study. In this study, explorative case descriptive design is used. There must be appropriate mentioning of among the well documented design. | The specific qualitative design applied in this study has now been clearly stated and included in methodology section |
| **6** | In line 81, it is well understood that an interview is done to a single person and therefore there is no need of stating that it is individual interview. | The individual has been removed since that does not change the meaning of the sentence |
| **7** | Methodology section is poorly arranged. For instance, it is not normal to mention recruitment of study participants before even mentioning study population. It is common to first have study design followed by description of study area, study population, sampling and then followed by recruitment of participants. This is not the case in this work hence confounding flow and logic. | The methodology section has been revised and rearranged to reflect the reviewer’s advice. |
| **8** | In my own understanding of your study, you have two study population which includes Nurses and caregivers. However, in your description from line 92-93, caregivers are not categorized as population but rather service users. In the following sentences, there is also evidence that caregivers are among study population. | This has been rectified. Caregivers have been appropriately classified as a study population. |
| **9** | Your sampling procedure is difficult to follow and thus can hardly be replicated elsewhere. In your sampling process, there is mentioning of levels of health facilities levels and numbers but there is no explanation on how these numbers were obtained from the facilities across study areas. | The sampling procedure has been clarified taking into consideration the reviewer’s comments. How the numbers of participants were obtained from the health facilities has been explained. |
| **10** | In line 107, there is mentioning of the status of individual which I think is not important in method section | This has been rectified |
| **11** | Data collection procedures do not show a place where interviews were carried out. In my own understanding a community is a group of families or households. In a situation like this where interviews are mentioned to have been done in community, it is difficult to know exact place of occurrence. One cannot tell if the interviews were conducted in household, church or at school compound. | The data collection procedure has been revised to show places where the interviews were conducted both in the health facilities and in the communities. |
| **12** | In line 123 there is merely mentioning of saturation. You must explain the process in which saturation was attained. This will also need to show parallel processes of data collection and analysis. | The process of data saturation has been explained now |
| **13** | Data management including quality control aspects are completely missing in the methodology section. | Data management including quality control has been added to the data analysis and the heading modified accordingly |
| **14** | In my understanding, listening of the audio recording is part of data management in particular, quality control process. What was the necessity of doing so after fieldwork? | This has been placed under quality control in data management |
| **15** | Data analysis approach is completely missing. There is no any approach such as thematic, content analysis and others forms, hence it is difficult to advise in absence of clear approach. However, there is mentioning of themes here but in a very simplified manner and cannot be replicated anywhere. This process must be well stipulated to enable the reader to understand the context in which the analysis was carried out. | The data analysis approach has been revised. Content analysis which is the approach adopted in this study has been stipulated to aid reader understanding of the context in which the analysis was done. |
| **16** | The section of methodology needs a major revision including rearrangement and improvement of the contents in most subsections | The methodology section has accordingly been revised and all suggestions incorporated. |
| **17** | RESULTS This section presents themes and sub-themes which emerged from an unknown analysis approach which is not mentioned in analysis sub-section. | The data analysis approach has been included to analysis sub-section to give meaning to the themes and sub-themes that emerged. |
| **18** | Line 189 presents a heading with new modified study attributes including health seeking behaviors which is not in your objective. You need to be consistent with terminologies used in this study. | The study attributes have been revised to be in line with the study objectives thus ensuring consistency with terminologies |
| **19** | A sub-theme number 3.1 needs to be health facility barrier and not in category of physical barrier | The sub-theme 3.1 has been moved to new theme and named appropriately as health facility barrier. |
| **20** | In presentation of data, you must introduce the quote. In this work, the quotes emerge abruptly without invitation of a reader to see the quote | The quotes have been introduced appropriately |
| **21** | Most of the quotes are product of translation and therefore I did not expect to see words such as don’t, can’t etc, since they can only be extracted from direct quote and not through translation. | The contracted forms in the quotes have been corrected. |
| **22** | From line 205-210, there are three quotes illustrating one thing. I think one of the quotes could suffice the need. This also applies to many parts of your results where too many quotes representing a single scenario are common. Some quotes are also not thoroughly analysed. | Some of the quotes have been removed |
| **23** | Looking at your quotes and their equivalent analysis paragraphs, the quotes are taking too many spaces as compared to analysis. In most cases, there are more than two quotes presenting single aspect. Under this manner, there is danger that analysis is compromised since some quotes grouped together presents different understanding. | Some of the quotes that convey the same information have been removed to avoid repetitions |
| **24** | You must be consistent in writing particularly your headings. Some of your headings are written in a way that all words are capitalizes while other are presented in lowercase. | This has been rectified |
| **25** | DISCUSSION Your discussion is written in a monotonous way. Some paragraphs are prefaced by the word “again it emerges” and “it further emerges”. This must be rectified. | The discussion section has been improved upon |
